# The Taiwan Birth Panel Study: a prospective cohort study for environmentally- related child health

**DOI:** 10.1186/1756-0500-4-291

**Published:** 2011-08-12

**Authors:** Chia-Jung Hsieh, Wu-Shiun Hsieh, Yi-Ning Su, Hua-Fang Liao, Suh-Fang Jeng, Feng-Ming Taso, Yaw-Huei Hwang, Kuen-Yuh Wu, Chia-Yang Chen, Yueliang Leon Guo, Pau-Chung Chen

**Affiliations:** 1 Institute of Occupational Medicine and Industrial Hygiene, National Taiwan University College of Public Health, Taipei, Taiwan; 2 Department of Pediatrics, National Taiwan University Hospital and National Taiwan University College of Medicine, Taipei, Taiwan; 3 Department of Medical Genetics, National Taiwan University Hospital and National Taiwan University College of Medicine, Taipei, Taiwan; 4 School and Graduate Institute of Physical Therapy, National Taiwan University College of Medicine, Taipei, Taiwan; 5 Department of Psychology, National Taiwan University College of Science, Taipei, Taiwan; 6 Institute of Environmental Health, National Taiwan University College of Public Health, Taipei, Taiwan; 7 Department of Environmental and Occupational Medicine, National Taiwan University Hospital and National Taiwan University College of Medicine, Taipei, Taiwan

**Keywords:** Cohort study, prenatal environmental exposure, child growth, neurodevelopment, behaviour problem, single-nucleotide polymorphisms

## Abstract

**Background:**

The Taiwan Birth Panel Study (TBPS) is a prospective follow-up study to investigate the development of child health and disease in relation to in-utero and/or early childhood environmental exposures. The rationale behind the establishment of such a cohort includes the magnitude of potential environmental exposures, the timing of exposure window, fatal and children's susceptibility to toxicants, early exposure delayed effects, and low-level or unknown neurodevelopmental toxicants.

**Methods:**

A total of 486 mother-infant paired was enrolled from April 2004 to January 2005 in this study. Maternal blood before delivery, placenta and umbilical cord blood at birth, and mothers' urine after delivery were collected. The follow-up was scheduled at birth, 4, 6 months, and 1, 2, 3 and 5 years. The children's blood, urine, hair, and saliva were collected at 2 years of age and children's urine was collected at 5 years of age as well. The study has been approved by the ethical committee of National Taiwan University Hospital. All the subjects signed the inform consent on entering the study and each of the follow up.

**Results:**

Through this prospective birth cohort, the main health outcomes were focused on child growth, neurodevelopment, behaviour problem and atopic diseases. We investigated the main prenatal and postnatal factors including smoking, heavy metals, perfluorinated chemicals, and non-persistent pesticides under the consideration of interaction of the environment and genes.

**Conclusions:**

This cohort study bridges knowledge gaps and answers unsolved issues in the low-level, prenatal or postnatal, and multiple exposures, genetic effect modification, and the initiation and progression of "environmentally-related childhood diseases."

## Background

Children's environmental health issues have been recognized by several international, national, and other organizations since the 1990s. A recent review stressed the roles of environmental factors on three groups of important childhood disorders, namely, asthma, childhood cancers, and neurodevelopmental disorders[1]. Environmental exposures include indoor and outdoor pollution, heavy metals, pesticides, and endocrine disrupting chemicals are important specific exposures for our children. Recognizing that exposure to hazardous environmental conditions can be particularly detrimental to the health of children, the National Institute of Environmental Health Sciences (NIEHS), the Environmental Protection Agency (EPA), and the Centers for Disease Control and Prevention (CDC) in the U.S. set up 12 Children's Environmental Health Centers in 1998 and 2000[2]. The environmentally-related childhood diseases targeted on respiratory diseases, growth and development, and neurodevelopment. The National Children's Study [3] has also been established according to the U.S. Children's Health Act of 2000. There were also large birth cohort studies in Denmark [4], Norway [5], and Japan [6]. The environmental-related child health issue has already raised large concerns worldwide.

Evidence is growing that prenatal and postnatal exposures and factors played a role in not only childhood but also adulthood disorders. There is a compelling need to undertake a prospective cohort study to evaluate the effects of such factors [7,8]. Several recent developments, including advancements in computer technology, the management, storage, and analysis of biological specimens, and the rapid growth of genetic markers, facilitate the evaluation of the influence of environmental exposures on the subsequent risk of developmental abnormalities and disease. The rationale behind the establishment of such a cohort includes the magnitude of potential environmental exposures, the timing of exposure window, fetal and children's susceptibility to toxicants, early exposure delayed effects, and low-level or unknown neurodevelopmental toxicants [9,10]. Due to lack of longitudinal birth cohort studies on the environment and child health in Taiwan, we set up a prospective birth panel, named Taiwan Birth Panel Study (TBPS), funded by the Bureau of Health Promotion, Department of Health in Taiwan during 2004-2005. Due to no further funding support from the Bureau of Health Promotion in Taiwan since 2006 we were seeking new research grants form National Science Council in Taiwan to support this prospective birth panel study to investigate the development of child diseases in relation to in-utero and/or early childhood environmental exposures during 2006-2012.

## Methods

### Study theme

**Figure 1:**
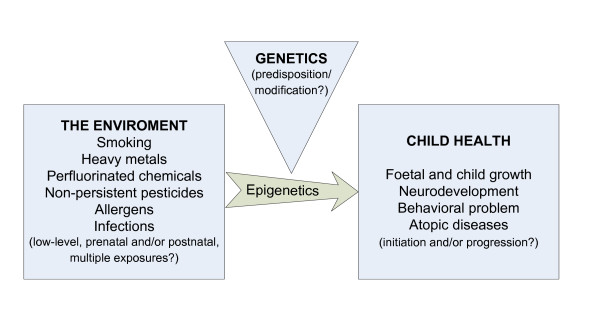
**The overview of the study topics of the Taiwan Birth Panel Study**.

The central theme of this cohort was to investigate prenatal and postnatal environmental factors on infant and early childhood health. Through a prospective birth cohort, the main health outcomes were focused on child growth, neurobehavioral development, and atopic diseases. We investigated the main prenatal and postnatal factors including smoking, heavy metals, perfluorinated chemicals, and non-persistent pesticides under the consideration of interaction of the environment and genes (Figure 1).

### Study area and participants

**Figure 2:**
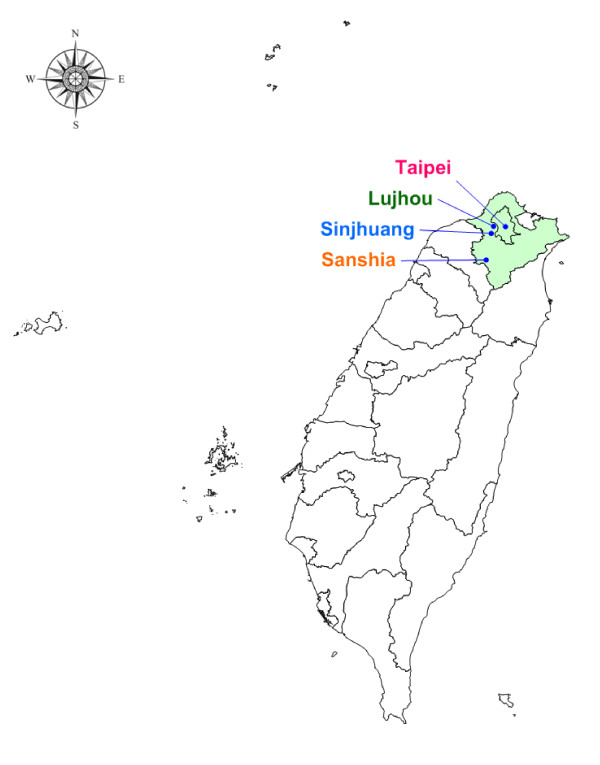
**Location of the TBPS recruitment centres**.

Considering potential environmental exposures in different socio-economic status, we recruited our subjects from one medical hospital in Taipei city (Taipei), one area hospital (Sansia), and two clinics in Taipei County (Sinjhuang, Lujhou) (Figure 2). In order to collect the mothers who had the similar characteristic distribution with the general population in Taiwan, we included all pregnant mothers who gave birth during the recruitment period and agreed to join this study. A total of 486 sets including both parents and their newborns were recruited between July 2004 and June 2005. The baseline socio-demographic characteristics of subjects in the TBPS study were shown in Table 1. In addition, the differences between a population-based, multistage stratified systematic sampling questionnaire survey in 2005 in Taiwan were also shown in Table 1. Comparing with the population-based survey, there were similar infant's characteristics but different maternal and family characteristics with the population-based survey. The subjects had higher maternal age and family income, than the population-based survey.

**Table 1:** Baseline socio-demographic characteristics of the subjects in the Taiwan Birth Panel Study

Characteristics, N (%)	TBPS study	Population-based survey
Total	486	21,248
**Mother's characteristics**		
Maternal age (%)		
<25 years	9.9	22.3
25-29 years	26.7	37.4
30-34 years	40.9	29.4
≥35 years	22.4	10.8
Maternal education (%)		
Junior high school and below	9.3	14.8
Senior high school	46.3	39.9
College and above	44.4	45.3
Ethnic origin		
Taiwanese	91.8	86.6
Foreign-born	8.2	13.4
Parity (%)		
Primipara	47.3	50.4
Multipara	52.7	46.6
**Family characteristics**		
Marital status (%)		
Married	97.5	97.1
Unmarried	2.5	2.8
Annual family income (%, NT$)		
< 600,000	27.6	41.7
600,000-1,000,000	32.5	36.6
1,000,000-1,500,000	24.5	14.5
≥ 1,500,000	15.4	7.2
**Infant's characteristics**		
Plurality (%)		
Singleton	98.8	97.4
Twin	1.2	2.6
Infant gender (%)		
Male	50.8	52.5
Female	49.2	47.5
Birth outcomes		
Gestational age (week, mean ± SD)	38.5 ± 1.7	38.4 ± 1.6
Birth weight (g, mean ± SD)	3157.9 ± 476.6	3104.2 ± 448.1

### Study design and follow ups

The TBPS study adopted a standard longitudinal design. A total of 486 mother-infant paired was enrolled from April 2004 to January 2005 in this study. Before enrolling the study participants, informed consents were obtained from mothers before delivery to collect maternal and umbilical cord blood and questionnaires. A structured questionnaire was interviewed within three days after delivery to collect parental demographics, socio-economic status, lifetime residential history, and environmental exposure history. For all the 486 sets, we had collected maternal blood before delivery (10 ml) and placenta (3*3*3) and umbilical cord blood (10 ml) at birth, mothers' urine (30 ml) after delivery. All the blood was added EDTA to avoid clotting and separated into whole blood, plasma and DNA. All the specimens were stored at -80°C until used.

The follow up was scheduled at birth, 4, 6 months, and 1, 2, 3 and 5 years. The mail-in questionnaires were used at the age of 4 months, 1 and 3 years. The face to face interview questionnaires were used at the age of 6 months and 2 and 5 years. In addition, the children's postnatal biological samples were collected the age of 2 and 5 years. The children's blood (5 ml), urine (25 ml), hair (2 g), and saliva (2 ml) were collected at 2 years of age and children's urine (25 ml) was collected at 5 years of age as well. Protocols used in these follow ups were approved by the Ethical Committee of National Taiwan University Hospital.

The summary of research works and timetable was shown in Additional file 1. Data on parental socio-demographic information, child growth, neurodevelopment, behaviour problem, atopic diseases, other common conditions, and potential environmental exposures was collected. The parental socio-demographic, child growth and postnatal environmental exposure information were collected at 4, 6 months, 1, 2, 3 and 5 years by structured questionnaires. The home visit was conducted to measure the quality and quantity of caregiving of the parents to a child in the home environment by using Infant/Toddler HOME of Home Observation for Measurement of the Environment Inventory (IT-HOME) [11] at 6 months and 2 years of age.

## Results

### Environmental exposure

The environmental exposure measurement was the main theme of TBPS study. The cotinine, heavy metals, perfluoroalkyl chemicals and non-persistent pesticides were analyzed.

### Cotinine

Cotinine, one of the most sensitive biomarker of nicotine for smoking exposure, was analyzed in maternal blood and cord blood by using high-performance liquid chromatography coupled to a triple-quadruple tandem mass spectrometer (HPLC-MS/MS).

### Heavy metals

Eighteen heavy metals were analyzed in maternal blood and cord blood by using inductively coupled plasma mass spectrometry (ICPMS), including lead, arsenic, mercury, cadmium, beryllium, antimony, barium, cerium, platinum, thorium, manganese, zinc, copper, selenium, cobalt, molybdenum, gallium and uranium.

### Perfluorinated chemicals

Twelve perfluorinated chemicals were analyzed by using ultraperformance liquid chromatography-tandem mass spectrometry (UPCL-MS/MS), including PFHxA, PFHpA, PFHxS, PFOA, PFOS, PFNA, PFDeA, PFUA, Me-PFOSA-AcOH, Et-PFOSA-AcOH, PFOSA, PFDoA in cord blood.

### Non-persistent pesticides

The organophosphate and pyrethroid pesticide was analyzed in cord blood by using online solid-phase extraction system coupled with liquid chromatography and a triple-quadrupole tandem mass spectrometer (Online SPE-LC/MS/MS), including chlorpyrifos, cypermethrin and flucythrinate.

### Outcomes of interested

#### Child growth

The Child Healthcare Handbooks was used to record the child height, weight and head circumference when they visited to clinics for health examinations or due to illness. The Taiwan National Health Insurance System offered the Child Healthcare Handbook for each child to record their health condition, vaccinations, growth and development before age of seven years. The information was measured by the doctor or nurse in each time to visit the clinic.

#### Neurodevelopment

The neurodevelopment was access at each follow up by different tools or questionnaires. The Neonatal Neurobehavioral Examination-Chinese Version (NNE-C) [12] was performed to detect the early children neurodevelopment by a trained physical therapist to access the need for a more quantitative assessment of neonatal neurobehavioral status in 3 days of age. The NNE-C scale consists of three parts: tone and motor patterns, primitive reflexes, and behavioral responses that each part contains nine items on a three-point scale. The information of child milestone was collected by using questionnaires at the age of 4, 6 month, 1, 2, and 3 years. In addition, a Comprehensive Developmental Inventory for Infants and Toddlers (CDIIT) test [13] was performed to detect the early childhood neurobehavioral performance at 6 months and 2 years. The CDIIT consists of cognitive, language, motor, social and self-help subtests. The MacArthur Communicative Development Inventory (CDI) [14] was used to detect the child language development at 2 years of age. To qualitative motor observations of children, the Movement Assessment Battery for Children - Second Edition (Movement ABC-2) [15] was used at the age of 5 years.

#### Behavioural problems

The Child Behavior Checklist/11/2-5 (CBCL/11/2-5) [16] was used to detect child's emotional and social functioning at 2, 3 and 5 years of age. The CBCL/11/2-5 features seven behavioral syndromes including emotionally reactive, anxious/depressed, somatic complaints, withdrawn, sleep problems, attention problems, and aggressive behavior. The former four syndrome scores were summed into the internalizing problem score; the latter two syndrome scores were summed into the externalizing problem score. The Chinese version of the Swanson, Nolan, and Pelham IV scale (SNAP-IV) [17] was used to detect child attention deficit hyperactivity disorder symptoms at the age of 5 years.

#### Temperament and Parenting Stress

The Toddler Temperament Scale [18,19] questionnaire was used to detect child temperament type at 4, 6 months, 1 and 2 years of age. The temperament types have been defined as easy, intermediate low, intermediate high, slow to warm up and difficult according to a suggested algorithm. Parenting Stress Index/short form (PSI/SF) [20] was used to detect the parenting stress at 2, 3 and 5 years of age. The PSI/SF contains three scales: Parental Distress, Difficult Child Characteristics, and Dysfunctional Parent-Child Interaction.

#### Serological markers of perinatal infections

Toxoplasmosis is caused by a protozoan parasite known as Toxoplasma gondii. Serum samples from cord blood of neonates and paired samples from their mothers were analyzed for Toxoplasma gondii-specific IgG and IgM titers by enzymelinked immunosorbent assay method (ELISA). Human cytomegalovirus (CMV) is a human-specific DNA virus that presents in various body fluids and transmits via interpersonal contact. The blood samples obtained from the paired maternal and cord bloods were tested by the ELISA method for CMV IgG and IgM antibodies.

#### Neuro-mediators as markers of allergic diseases

For atopic disease, the neuro-mediators were taken as predictors of atopic dermatitis. The immunoglobulin E (IgE) was analyzed in maternal blood, cord blood and child blood at age 2, and nerve growth factor (NGF) and vaso-active intestinal peptide (VIP) in cord and maternal plasma were analyzed.

### Single-nucleotide polymorphisms (SNPs)

#### Smoking related SNPs

Genes reported to play an important role in detoxifying smoke-derived toxic chemicals through metabolism and other detoxification processes were analyzed by using real-time PCR on a lightCyclerTM 480 instrument (Roche, Mannheim, Germany) in maternal and cord blood DNA, including CYP1A1 Msp1, CYP1A1 Ile462Val, GSTT1, GSTM1 and GSTP1 gene.

#### Heavy metals related SNPs

The superoxide dismutase 2 (SOD2) genes, SOD2 Val16Alam and SOD2 -102C>T, were analyzed as well, because the manganese is a component of the antioxidant enzyme and SOD2 is involved in controlling dioxygen toxicity in mitochondria, and protects cells from oxidative stress. Moreover, to study the gene modification effect of mercury, three apolipoprotein E (APOE) alleles code for isoforms E2, E3, and E4 were analyzed in this study. Based on the different amino-acid configurations, the isomers and their potential relevance may affect the mercury elimination. The vitamin D receptor (VDR) genotype can modify the adsorption of lead from the uptake and release of lead form bone. The δ-aminolevulinic acid dehydratase (ALAD) gene can modify pharmacokinetic distribution of lead. The VDR FokI, VDR BsmI and ALAD were analyzed by using polymerase chain reaction-restriction fragment length polymorphism (PCR-RFLP).

#### Pesticides related SNPs

The paraoxonase-1 (PON1) is an enzyme involved in the metabolism of certain organophosphate pesticides. The PON1 gene is a gene that regulates the activity and expression of PON1. For this reason, the PON1 L55M, PON1 Q192R and PON1 C(-162)T were analyzed by using real-time PCR on a lightCyclerTM 480 instrument (Roche, Mannheim, Germany).

## Discussion

Due to the main themes of TBPS study was to explore the potential health effect of environmental exposures and fatal and children's susceptibility to toxicants, we had published on the smoking, heavy metals, and psychosocial factors and the potential gene modification effect on child health as well as infections and markers of allergic diseases. The detailed publication list was put on the TBPS website (http://www.rhlab.org/tbps.html).

In summary, we found that prenatal exposure may be associated with adverse child health, including child neurodevelopment, behavioral problem, growth and atopic dermatitis in early childhood. These findings were focused on environmental tobacco smoke, heavy metals, maternal mental health and stress (table 2).

**Table 2:** Measurements in blood sample in the Taiwan Birth Panel Study

Environmental exposures	Specimen	Measures
Smoking	Maternal and cord blood	Cotinine
Heavy metals [25-27]	Maternal and cord blood	Pb, As, Hg, Cd, Be, Sb, Ba, Ce, Pt, Th, Mn, Zn, Cu, Se, Co, Ga, Mo, U
Perfluorinated chemicals	Cord blood	PFHxA, PFHpA, PFHxS, PFOA, PFOS, PFNA, PFDeA, PFUA, Me-PFOSA-AcOH, Et-PFOSA-AcOH, PFOSA, PFDoA
Non-persistent pesticides	Cord blood	Organophosphates: chlorpyrifos. Pyrethroids: cypermethrin, flucythrinate.
**Diseases**		
Atopic diseases [23,24]	Maternal, cord blood and child blood at age 2	IgE
	Maternal and cord blood	NGF,VIP
Infection [30,31]	Maternal and cord blood	IgM & IgG of toxoplasmosis and cytomegalovirus
**Genotyping**		
Single-nucleotide polymorphisms		
Smoking [21,22]	Maternal and cord blood DNA	CYP1A1 Msp1, CYP1A1 Ile462Val, GSTT1, GSTM1, GSTP1
Pesticides	Maternal and cord blood DNA	Paraoxonase-1 (PON1) L55M, paraoxonase-1 (PON1) Q192R, paraoxonase-1 (PON1) C(-162)T
Mercury	Cord blood DNA	Apolipoprotein E (APOE)
Manganese	Cord blood DNA	Manganese superoxide dismutase 2 (MnSOD2)
Lead	Cord blood DNA	Vitamin D receptor BsmI (VDR BsmI), Vitamin D receptor FokI (VDR FokI), δ-aminolevulinic acid dehydratase (ALAD)
DNA methylation	Cord blood DNA	Illumina methylation array

Firstly, we found infant genes can modify prenatal environmental tobacco smoke on early children's neurodevelopmental and behavioral problems [21,22]. In addition, smoke exposure during pregnancy might increase the risk of atopic dermatitis in children [23]. The genetic polymorphisms in GSTM1 and GSTP1 may be responsible for differences in susceptibility to atopic dermatitis with regard to prenatal smoke exposure [24].

Secondly, we established the concentration distributions of heavy metals in umbilical cord blood in Taiwan [25] and found the relationship between cord blood manganese levels and fine-motor showed an inverted U shape [26]. In addition, cord blood lead was lower where the mother had a higher blood concentration of zinc or manganese [27]. This finding raises the possibility of reducing placental transfer of lead by increasing zinc levels via nutritional supplementation during pregnancy.

Thirdly, the potential effect of maternal psychosocial factors, mental health and working stress on child neurodevelopment and behaviour was also studied. We found maternal vitality around delivery was beneficial to a child's self-help development, while work stress seemed to be adverse effect on child's motor development in later life [28]. In addition, working stress around delivery seems to aggravate child's externalizing behaviour problems at two years old [29].

Finally, our study findings have highlighted the emerging importance of current perinatal cytomegalovirus (CMV) as an important perinatal infection among immigrant mothers and teenage mothers, and high servoprevalence of CMV in Taiwan [30,31].

### Strengths and weaknesses

Due to lack of longitudinal birth cohort studies on the environment and child health in Taiwan, little epidemiological information is available to explore the issue. The TBPS is population-based, biological samples collected and regular follow ups to elucidate the effects of in-utero and/or postnatal environmental exposure and to explore the interaction effect between gene and environment. Therefore, we can achieve clear causal relationships without recall bias as well as potential measurement errors.

The main limitation of this study is that we recruited the subjects before delivery, as the reason, we lack detailed information during the different trimester of pregnancy and cannot collect biological sample to analyze the exposure condition in different trimesters. In addition, we couldn't answer the rare diseases or health outcomes due to our modest sample size.

## Conclusion

This cohort study bridges knowledge gaps and answers unsolved issues in the low-level, prenatal or postnatal, and multiple exposures, genetic effect modification, and the initiation and progression of "environmentally-related childhood diseases." All the data are held by the Taiwan Birth Panel Study team at National Taiwan University College of Public Health and National Taiwan University Hospital. External collaboration is welcomed. We had set up a website (http://www.rhlab.org/tbps.html). Moreover, further information about the study can be contact Prof. Pau-Chung Chen at http://pchen@ntu.edu.tw.

## List of abbreviations

TBPS: Taiwan Birth Panel Study; SNPs: Single-nucleotide polymorphisms; IT-HOME: Infant/Toddler HOME of Home Observation for Measurement of the Environment Inventory; NNE-C: Neonatal Neurobehavioral Examination-Chinese Version; CDIIT: Comprehensive Developmental Inventory for Infants and Toddlers; CBCL/11/2-5: Child Behavior Checklist/11/2-5; CDI: MacArthur Communicative Development Inventory; Movement ABC-2: Movement Assessment Battery for Children - Second Edition; SNAP-IV: Swanson, Nolan, and Pelham IV scale; PSI/SF: Parenting Stress Index/short form; IgE: Immunoglobulin E; NGF: Nerve growth factor; VIP: Vaso-active intestinal peptide.

## Competing interests

The authors declare that they have no competing interests.

## Authors' contributions

PC, WS, and YL designed the study. CJ performed the data analysis and wrote the manuscript. KY, YH and CY supervised the specimen's analysis. YN provided support with DNA analysis. HF, SF and FM carried out the neurodevelopment measurement. All authors have read and approved the final manuscript.

## Supplementary Material

Additional file 1 **The summary of study design and the timetable**. Click here for file
