# Supplementary material for: The Taiwan Birth Panel Study: a prospective cohort study for environmentally- related child health
Source: BMC Res Notes. 2011 Aug 12;4:291. doi: 10.1186/1756-0500-4-291 (PMC3170609; doi:10.1186/1756-0500-4-291)
Supplement: Additional file 1 — The summary of study design and the timetable. [file 1756-0500-4-291-S1.DOC]

**Table. The summary of study design** and the timetable

|  | 2004 | | | 2005 | | | 2006 | | 2007 | | 2008 | 2009 | | 2010 | |  |
| --- | --- | --- | --- | --- | --- | --- | --- | --- | --- | --- | --- | --- | --- | --- | --- | --- |
| Funding support | Bureau of Health Promotion | | | | | National Science Council | | | | | | | | | | |
| Field works/ methods |  | • Phase-0  Mother-newborn recruitment | | | |  | | | | | | | | | | |
|  |  | | • Phase-1  4-month-old follow-up | | • Phase-2  6-month-old follow-up | | | • Phase-3  1-year-old follow-up | | • Phase-4  2-year-old follow-up | | | • Phase-5  3-year-old follow-up | | • Phase-6  5-year-old follow-up | |
| Specimen | • Maternal blood before delivery, placenta & DNA  • Cord blood & DNA  • Maternal urine | | | |  | | |  | | • Child blood, urine, hair & saliva | | |  | | • Child urine | |
| Home visit |  | | | | • HOME score | | |  | | • HOME score | | |  | |  | |
| Socio-economic/ environmental questionnaires | • Lifestyle self-reported questionnaire | | | | • Lifestyle self-reported questionnaire | | | • Lifestyle self-reported questionnaire | | • Lifestyle self-reported questionnaire | | | • Lifestyle self-reported questionnaire | | • Lifestyle self-reported questionnaire | |
| Child growth & health | • Medical record abstract  • Foetal growth  • Foetal health | | | | • Foetal growth  • Atopic diseases  • Foetal health | | | • Child growth  • Atopic diseases  • Child health | | • Child growth  • Atopic diseases  • Child health | | | • Child growth  • Atopic diseases  • Child health | | • Child growth  • Atopic diseases  • Child health | |
| Neurodevelopment  & Behaviour problem | • Milestone  • Neonatal neurobehavioural development | | | | • Milestone  • Neurodevelopment | | | • Milestone | | • Milestone  • Neurodevelopment  • Behaviour problem  • Language development | | | • Behaviour problem | | • Motor development  • Behaviour problem  • Attention deficit/ hyperactivity disorder | |
| Temperament/  Stress | • Neonatal temperament | | | | • Neonatal temperament | | | • Neonatal temperament | | • Child temperament  • Parenting stress index  • Child motivation | | | • Parenting stress index | | • Parenting stress index  • Child motivation | |
